# The prevalence of malignant and borderline ovarian cancer in pre- and post-menopausal Chinese women

**DOI:** 10.18632/oncotarget.20384

**Published:** 2017-08-22

**Authors:** Fang Shen, Shouzhen Chen, Yifei Gao, Xujing Dai, Qi Chen

**Affiliations:** ^1^ The Hospital of Obstetrics and Gynaecology, Fudan University, China; ^2^ Department of Obstetrics and Gynaecology, The University of Auckland, New Zealand

**Keywords:** ovarian cancer, menopause, age at diagnosis, Chinese women

## Abstract

The incidence of ovarian cancer depends on the ethnicity and geographical area. Menopausal status is a well-known risk factor for ovarian cancer and most cases occur after menopause in Caucasians. However, it is less clear how the status of menopause or age at diagnosis is associated with ovarian cancer including its subtypes in Chinese women. Data on 1,283 women with primary malignant or borderline ovarian cancer including age at diagnosis, age at menopause and histology from the largest women's hospital in China was analysed. The median age at diagnosis was 53, 44 and 23 years for epithelial ovarian cancer or sex-cord tumors or germ cell tumors respectively. 58% of epithelial ovarian cancers were diagnosed after menopause, while 58% and 95% of sex-cord tumors and germ cell tumors were diagnosed before menopause. Around 60% of serous, endometrioid and clear-cell carcinoma of epithelial ovarian cancer were diagnosed after menopause, while 23% of mucinous carcinoma was diagnosed after menopause. The median age at diagnosis was 35 years for borderline ovarian cancer and 80% of patients were diagnosed before menopause.

Our data demonstrates that the median age at diagnosis of ovarian cancer is younger in our study population than Caucasians reported in the literature regardless of malignant or borderline ovarian cancers, and regardless of subtypes of epithelial ovarian cancer. The prevalence of ovarian cancer is proportionately higher in our study population before menopause than Caucasians. Our results suggest clinicians to be more aware of the symptoms of ovarian cancer in premenopausal Chinese women.

## INTRODUCTION

Ovarian cancer is a major gynaecological cancer with more than 220,000 newly diagnosed cases every year in developed countries in 2014 and is the 5th leading cause of death in women worldwide [1]. The exact causes of ovarian cancer are still unclear, which results in no screening test. However a number of risk factors which are associated with the changes in levels of sex hormones during women's lifetime, for developing ovarian cancer have been identified. These risk factors include early menarche and late menopause, the number of ovulation, age at menopause, obesity, hormone replacement treatment (HRT) during menopause and ethnicity [2–7].

Increasing a woman's exposure to high levels of circulating estrogen likely increases the risk of developing ovarian cancer although the association between HRT and the incidence of ovarian has not been fully confirmed by studies with large sample size [8]. Before menopause, the ovaries are the major source for estrogen and progesterone production. In addition, estrogens are also produced through peripheral aromatization of testosterone, especially in obese women [9], which suggests that obesity increases the risk of developing ovarian cancer, but this risk is not present after menopause [10, 11]. After menopause, the ovaries stop synthesising estrogen and progesterone. A shift in the balance of these two sex hormones towards more estrogen increases the risk for developing ovarian cancer. A study suggested that an early age at onset of menopause is negatively correlated with developing ovarian cancer [12]. Collectively these studies suggested that menopause at older age is one of the risk factors for ovarian cancer. However, studies about the association of menopause with the prevalence of ovarian cancer in Chinese women are limited.

A number of studies reported that the prevalence and the survival of gynaecological cancers including ovarian cancer depend on the ethnicities and geographical areas (world caner report 2014). Asian women have lowest incidence of ovarian cancer (review in [13]) and normally have better clinical outcomes compared to North American and European (world caner report 2014), which are similar to endometrial cancer, another endocrine related cancer [14]. Globally, the majority of ovarian cancer is most commonly diagnosed after menopause between the ages of 60 to 64 years, with the typical age of 63 years at diagnosis (Cancer Research UK). Furthermore, 90% of ovarian cancer occurs in women over the age of 45 and 80% in women over 50 years (Cancer Research UK). However, study reported that the age at diagnosis of endometrial cancer in Asian women, including Asian native and Asian immigrants, is younger than non-Asian women [14]. Our recent studies also showed that the median age at diagnosis of endometrial cancer was 55 years old in Chinese women, who is younger than other ethnicities in developed countries and around 40% of Chinese women with endometrial cancer were diagnosed before menopause [15, 16]. Our unpublished preliminary data reported that the age at diagnosis with malignant ovarian cancer was 53 years in Chinese women. The difference in the age at diagnosis could be due to the effect of ethnicity on hormonal and cancer characteristics including histological subtypes [17, 18].

Ovarian cancers are traditionally classified into three main subtypes including epithelial ovarian carcinoma, sex cord stromal tumors and germ cell tumors based on presumed histogenesis and direction of differentiation. Each of these three classifications also includes a series of subtypes [19]. Epithelial ovarian carcinoma accounts for about 90% of all ovarian cancers and includes four subtypes: serous, mucinous, endometrioid and clear-cell carcinoma. Sex-cord tumors and germ cell tumors are less common, which account for about 7% or 5% of all ovarian cancers, respectively.

Whether Asian (Chinese) women having better clinical outcomes of ovarian cancer associated with the age at diagnosis or the status of menopause has not been fully investigated. In this study, we performed a retrospective analysis of data obtained from the largest university Obstetrics and Gynaecology teaching hospital serving diverse urban and rural areas in China to investigate the association between subtypes of ovarian cancer, age at diagnosis and menopause in our study population.

## RESULTS

### The prevalence of diagnosed malignant ovarian cancer between premenopausal and postmenopausal women

The clinical characteristics of patients with malignant ovarian cancer (*n* = 1,016) are summarised in Table 1 based on the subtypes of cancer. Because the ages of subtypes of ovarian cancer at diagnosis are different, we firstly divided ovarian cancer into epithelial ovarian cancer, sex-cord tumors and germ cell tumors to analyse the age distribution of cancers (Table 1). The median age of patients with malignant epithelial ovarian cancer, or sex-cord tumors or germ cell tumors was 53 (range 17–79) years, or 44 (range 16–70) years, or 23 (14–65) years, respectively. Of 854 patients with epithelial ovarian cancer, 361 (42%) patients were diagnosed before menopause and 493 (58%) patients were diagnosed after menopause. The prevalence of diagnosed epithelial ovarian cancer was significantly higher in postmenopausal women compared to premenopausal women (*p* < 0.0001).

**Table 1:** Clinical parameters of malignant ovarian cancer according to subtypes

	Epithelial ovarian cancer (*n* = 871)	Sex-cord tumors (*n* = 86)	Germ cell tumors (*n* = 59)
Age at diagnosis (years, median/range)	53 (17–79)	44 (16–70)	23 (14–65)
Premenopause (*n*, column %)	361(42%)	50 (58%)	56 (95%)
Postmenopause (*n*, column %)	493 (58%)	36 (42%)	3 (5%)
Age of premenopausal women (years, median/range)	44 (17–58)	33 (16–54)	23 (14–48)
Age of postmenopausal women (years, median/range)	58 (43–79)	57 (41–70)	53 (52–65)

17 women from epithelial ovarian cancer had hysterectomy resulting in no information about their menopausal status.

Of 86 patients with sex-cord tumors, 50 (58%) patients were diagnosed before menopause and 36 (42%) patients were diagnosed after menopause. Of 59 patients with germ cell tumors, 56 (95%) patients were diagnosed before menopause and 3 (5%) patients were diagnosed after menopause. The prevalence of diagnosed sex-cord tumors or germ cell tumors was significantly higher in premenopausal women compared to postmenopausal women (*p* < 0.0001).

### The prevalence of diagnosed subtypes of epithelial ovarian cancer between premenopausal and postmenopausal women

We then analysed the prevalence of subtypes of epithelial ovarian cancer according to menopausal status (Table 2). The prevalence of diagnosed serous carcinoma, endometrioid carcinoma and clear-cell carcinoma was significantly higher in postmenopausal women compared to premenopausal women (62%, 57%, 59%, respectively, *p* < 0.0001). While the prevalence of diagnosed mucinous carcinoma was significantly higher in premenopausal women (77%) compared to postmenopausal women (23%) (*p* < 0.0001).

**Table 2:** Clinical parameters of subtypes of malignant epithelial ovarian cancer

	Serous carcinoma (*n* = 587)	Mucinous carcinoma (*n* = 64)	Endometrioid carcinoma (*n* = 60)	Clear-cell carcinoma (*n* = 160)
Age at diagnosis (years, median/range)	54 (23–79)	43 (17–73)	53 (28–76)	51(29–70)
Premenopause (*n*, column %)	222 (38%)	48 (77%)	25 (43%)	64 (41%)
Postmenopause (*n*, column %)	356 (62%)	14 (23%)	33 (57%)	92 (59%)

9 women from serous carcinoma, 2 women from mucinous carcinoma, 2 women from endometrioid carcinoma and 4 women from clear-cell carcinoma had hysterectomy resulting in no information about their menopausal status.

### The prevalence of diagnosed borderline ovarian cancer between premenopausal and postmenopausal women

The clinical characteristics of patients with borderline ovarian cancer (*n* = 267) were summarised in Table 3. The median age of patients at diagnosis was 35 (range 13–85) years. Of 267 patients, 213 (80%) patients were diagnosed before menopause and 52 (20%) patients were diagnosed after menopause. The prevalence of diagnosed borderline ovarian cancer was significantly higher in premenopausal women compared to postmenopausal women (*p* < 0.0001).

**Table 3:** Clinical parameters of borderline ovarian cancer

	Total number of patients (*n* = 267)
Age at diagnosis (years, median/range)	35 (13–85)
Premenopause (*n*, column %)	213 (80%)
Postmenopause (*n*, column %)	52 (20%)
Age of premenopausal women (years, median/range)	32 (13–55)
Age of postmenopausal women (years, median/range)	62 (48–85)

2 women from borderline ovarian cancer had hysterectomy resulting in no information about their menopausal status.

### Age distribution of patients with malignant and borderline ovarian cancer at diagnosis

We further analysed the age distribution of malignant ovarian cancer at diagnosis according to three subtypes of ovarian cancer. In cases of epithelial ovarian cancer, 674 (77%) patients were diagnosed after 45 years and 545 (62%) patients were diagnosed after 50 years. In cases of sex-cord tumors, 31 (36%) patients were diagnosed after 50 years. In cases of germ cell tumors, 44 (75%) patients were diagnosed before 30 years.

We also analysed the age distribution of borderline ovarian cancer at diagnosis. 161 (60%) patients were diagnosed before 40 years which was significantly higher than patients (40%) who were over 40 years (*p* < 0.0001).

## DISCUSSION

The incidence of gynaecological cancers including ovarian cancer depends on the ethnicities and geographical areas (world caner report 2014) [17, 18] and the incidence of ovarian cancer is recently significantly increased in China since last decade [20]. In general, the majority of malignant ovarian cancer is most commonly diagnosed after menopause between the ages of 60 to 64 years, with the typical age of 63 years at diagnosis in Caucasians (Cancer Research UK). In our current retrospective study, data were collected from the largest obstetrics & gynaecology hospital in China during 6 years study period with 1,283 cases and we report that the median age at diagnosis of malignant epithelial ovarian cancer was 53 (range 17–79) years, which is approximately 10 years younger than Caucasians reported in the literature. The majority of sex-cord tumors commonly occurs in women between 50–70 years (Cancer Research UK), with the average age of 50 years at diagnosis in the United States [21], but in our current study we found that the median age at diagnosis of sex-cord tumors was 44 (range 16–70) years which is also approximately 6 years younger than Caucasians reported in the literature. The majority of germ cell tumors (70%) commonly occur in young women between 20–30 years in Caucasians. In this current study we found that 75% cases of germ cell tumors occurred before 30 years which is similar with other studies.

Cancer Research (UK) reported that 90% of epithelial ovarian cancer occurs over the age of 45 and 80% of epithelial ovarian cancer occurs over the age of 50 years in Caucasians. A Sweden study also found that 86% of ovarian cancer were diagnosed over the age of 40 years [22]. However in our current study, we found 77% of epithelial ovarian cancer was diagnosed over the age of 45 years in our study population which is 10%-15% lower than Caucasians reported in the literature. Furthermore, only 62% of epithelial ovarian cancer was diagnosed over the age of 50 years, which is also 20% lower than Caucasians reported in the literature. Among the cases of sex-cord tumors, the Surveillance Epidemiology and End Results (SEER) United States data showed 12% of cases were younger than 30 years [21]. However in our current study we found that 20% of cases of sex-cord tumors were younger than 30 years, 64% of cases were younger than 50 years in our study population, which are higher than Caucasians. However the effect of younger age at diagnosis was not seen in germ cell tumors, compared to other subtypes of ovarian cancer. Taken together our data suggest that the age at diagnosis of epithelial ovarian cancer or sex-cord tumors was younger in our study population than Caucasians reported in the literature.

Menopausal status, late onset of menopause is one of the risks of developing epithelial ovarian cancer (world caner report 2014). Studies reported that most of cases of epithelial ovarian cancer occur after menopause in Caucasians. A recent multicentre case-control study also showed that 73% of epithelial ovarian cancer occurred after menopause in the United States [23]. However, in our current study we found that the prevalence of epithelial ovarian cancer in our study population was slightly higher in postmenopausal women (58%) than in premenopausal women (42%), but this prevalence of epithelial ovarian cancer in postmenopausal women in our study population (58%) at the time of diagnosis is still lower than that in Caucasians reported in the literature [23]. In contrast, the prevalence of epithelial ovarian cancer in premenopausal women is higher in our study population. We do not know the exact reason for this difference, but this could be due to the effect of ethnicity on hormonal and cancer characteristics including histological subtypes [17, 18].

Borderline ovarian cancer comprises around 15–20% of all epithelial ovarian cancer [22, 24], and it usually affects women aged between 20 and 40 and, 80% of cases are usually diagnosed at an early stage (Cancer Research UK) [25]. However, a Sweden study reported the median age at diagnosis of borderline ovarian cancer was 55.2 years [22]. Other two studies reported that the median age at diagnosis of borderline ovarian cancer was 45 years in Caucasians [26, 27]. In our current study, we found that the median age at diagnosis of borderline ovarian cancer was 35 (range 13 to 85) years in our study population, which is more than 10 years younger than other ethnicities. Although one third of cases were diagnosed younger than 40 years in Caucasians (review in [28, 29]), our data showed that 60% of cases were diagnosed younger than 40 years in our study population. Taken together our data suggest that age at diagnosis of borderline ovarian cancer is also younger in our study population than Caucasians.

There are some limitations to this study. First, the age of menopause was self-reported. Second, data were collected from a single Obstetrics & Gynaecology university teaching hospital in China, and we acknowledge that the age at menopause may vary among regions and economic levels in China. Third, we do not have data available on BMI, history of hypertension and diabetes which are risk factors for ovarian cancer, however to date there is no evidence indicating these risk factors are associated with the time of onset of ovarian cancer. Future study may be required. Other information such as family history of ovarian cancer, parity and history of abortion were also not available for all the patients.

In conclusion, in this large retrospective study we found that the median age at diagnosis of ovarian cancer is younger in our study population than Caucasians reported in the literature regardless of subtypes of malignant (epithelial ovarian cancer and sex-cord tumors) or borderline ovarian cancers, and regardless of subtypes of epithelial ovarian cancer (excluding mucinous carcinoma). The prevalence of ovarian cancer is proportionately higher in our study population before menopause than Caucasians reported in the literature. Our results strongly recommended clinicians to be more aware of the symptoms of ovarian cancer such as bloating, abdominal or pelvic pain or abnormal bleeding in premenopausal Chinese women.

## MATERIALS AND METHODS

This study was approved by the Ethics Committee of The Hospital of Obstetrics & Gynaecology, Fudan University, China for use of the hospital anonymised database.

### Study participants

This retrospective data was collected from the largest Obstetrics and Gynaecology university teaching hospital, The Hospital of Obstetrics and Gynaecology, Fudan University, which serve a diverse urban and rural population in China. In this study, data on 1,283 women with primary ovarian cancer were collected according to the electronic based medical records of patients from January 2010 to December 2015. Of 1,283 patients, 1,016 patients were primarily diagnosed with malignant ovarian cancer and 267 patients were primarily diagnosed with borderline ovarian cancer. Clinical characteristics included age at diagnosis, self-reported age at menopause, and pathological findings.

A diagnosis of ovarian cancer is confirmed through a surgical specimen, following removal of ovary during surgery. The classification of ovarian cancer was determined by pathological examination of surgical specimen, including cancer histologic subtype and grade. According to the classification of the International Federation of Gynaecology and Obstetrics (FIGO), malignant ovarian cancers are divided into epithelial ovarian cancer, sex-cord tumors and germ cell tumors. We also further classified epithelial ovarian cancer to serous carcinoma, mucinous carcinoma, endometrioid carcinoma and clear-cell carcinoma. Borderline ovarian cancers, as a heterogeneous group of lesions are defined histologically by increased epithelial proliferation accompanied by nuclear atypias without stromal invasion [30]. The clinical and histological characteristics of malignant ovarian cancer and borderline epithelial ovarian cancer were summarized in Tables 1 and 3.

### Statistical analysis

The statistical difference in the number of patients with epithelial ovarian cancer or sex-cord tumors or germ cell tumors or borderline ovarian cancer between premenopausal and postmenopausal women was assessed by the proportion test using the Prism software package. *p* < 0.05 was considered statistically significant.
